# Genotype Diversity before and after the Introduction of a Rotavirus Vaccine into the National Immunisation Program in Fiji

**DOI:** 10.3390/pathogens10030358

**Published:** 2021-03-17

**Authors:** Sarah Thomas, Celeste M. Donato, Sokoveti Covea, Felisita T. Ratu, Adam W. J. Jenney, Rita Reyburn, Aalisha Sahu Khan, Eric Rafai, Varja Grabovac, Fatima Serhan, Julie E. Bines, Fiona M. Russell

**Affiliations:** 1 Enteric Diseases Group, Murdoch Children’s Research Institute, Parkville, VIC 3052, Australia; celeste.donato@mcri.edu.au (C.M.D.); jebines@unimelb.edu.au (J.E.B.); 2 Department of Paediatrics, The University of Melbourne, Parkville, VIC 3052, Australia; 3 Ministry of Health and Medical Services, Suva, Fiji; soccovea@gmail.com (S.C.); tupou.ratu@gmail.com (F.T.R.); aalisha@gmail.com (A.S.K.); eric.rafai@govnet.gov.fj (E.R.); 4 Asia-Pacific Health Group, Murdoch Children’s Research Institute, Parkville, VIC 3052, Australia; jenneya@unimelb.edu.au (A.W.J.J.); buaha@gmail.com (R.R.); fmruss@unimelb.edu.au (F.M.R.); 5 College of Medicine, Nursing and Health Sciences, Fiji National University, Suva, Fiji; 6 Centre for International Child Health, Department of Paediatrics, The University of Melbourne, Parkville, VIC 3052, Australia; 7 Western Pacific Regional Office, World Health Organization, Manila 1000, Philippines; grabovacv@who.int; 8 World Health Organization, 1202 Geneva, Switzerland; serhanfa@who.int; 9 Department of Gastroenterology and Clinical Nutrition, Royal Children’s Hospital, Parkville, VIC 3052, Australia

**Keywords:** rotavirus, Fiji, Rotarix, genotype, equine-like G3P[8]

## Abstract

The introduction of the rotavirus vaccine, Rotarix, into the Fiji National Immunisation Program in 2012 has reduced the burden of rotavirus disease and hospitalisations in children less than 5 years of age. The aim of this study was to describe the pattern of rotavirus genotype diversity from 2005 to 2018; to investigate changes following the introduction of the rotavirus vaccine in Fiji. Faecal samples from children less than 5 years with acute diarrhoea between 2005 to 2018 were analysed at the WHO Rotavirus Regional Reference Laboratory at the Murdoch Children’s Research Institute, Melbourne, Australia, and positive samples were serotyped by EIA (2005–2006) or genotyped by heminested RT-PCR (2007 onwards). We observed a transient increase in the zoonotic strain equine-like G3P[8] in the initial period following vaccine introduction. G1P[8] and G2P[4], dominant genotypes prior to vaccine introduction, have not been detected since 2015 and 2014, respectively. A decrease in rotavirus genotypes G2P[8], G3P[6], G8P[8] and G9P[8] was also observed following vaccine introduction. Monitoring the rotavirus genotypes that cause diarrhoeal disease in children in Fiji is important to ensure that the rotavirus vaccine will continue to be protective and to enable early detection of new vaccine escape strains if this occurs.

## 1. Introduction

Rotavirus is the most common cause of severe diarrhoea in children under 5 years of age worldwide. In 2016, rotavirus was responsible for 258 million episodes of diarrhoea and was attributed to ~128,500 deaths in children under 5 years, with the majority occurring in countries in Asia and Africa [1]. Genotyping of rotavirus strains underpins global rotavirus surveillance. The binomial classification of rotavirus genotypes is based on the outer capsid proteins VP7 and VP4 that define G and P genotypes, respectively [2]. There are 36 G types and 51 P types described in humans and various animal species to date; however, the most common rotavirus genotypes observed in humans are the VP7 genotypes: G1, G2, G3, G4 and G9 and the VP4 genotypes: P[4] and P[8], representing three quarters of all genotypes causing human disease [3,4]. Previously uncommon genotypes including G12 and equine-like G3P[8] genotypes are increasingly being identified as a cause of rotavirus disease globally [5,6].

Fiji is a Pacific Island Nation with a population of approximately 837,271 [7]. Although designated as an upper middle-income country, it was estimated that prior to the COVID-19 pandemic, 24% of the population were living in poverty [7]. The child under-5-year mortality rate in Fiji was reported as 25.7 deaths per 1000 live births in 2019 [8]. Rotavirus was a major cause of diarrhoea-related hospitalisations in Fiji prior to rotavirus vaccine introduction, detected in 52% (2006) and 60% (2007) of children less than 5 years hospitalised with acute diarrhoea, with an annual incidence estimated at 486 per 100,000 children less than 5 years [9]. Due to this burden of rotavirus gastroenteritis, Fiji introduced a rotavirus vaccine (Rotarix, GlaxoSmithKline, Belgium) into the National Immunisation Program in October 2012. Rotarix is a monovalent vaccine containing a single, human, G1P[8] strain that is administered in a two-dose schedule at 6 and 14 weeks of age. The uptake of Rotarix in Fiji was prompt, reaching 85% coverage by 2013 and 99% coverage in eligible infants from 2014 onward [10]. The introduction of rotavirus vaccines in Fiji has been highly successfully resulting in an 82% reduction in rotavirus diarrhoea related hospitalisations in children less than 5 years of age [11].

The aim of this study was to describe the pattern of rotavirus genotype diversity from 2005 to 2018, specifically to describe any changes in genotype patterns that may have occurred following the introduction of the rotavirus vaccine in Fiji in 2012.

## 2. Results

### 2.1. Study Samples

**Figure 1:**
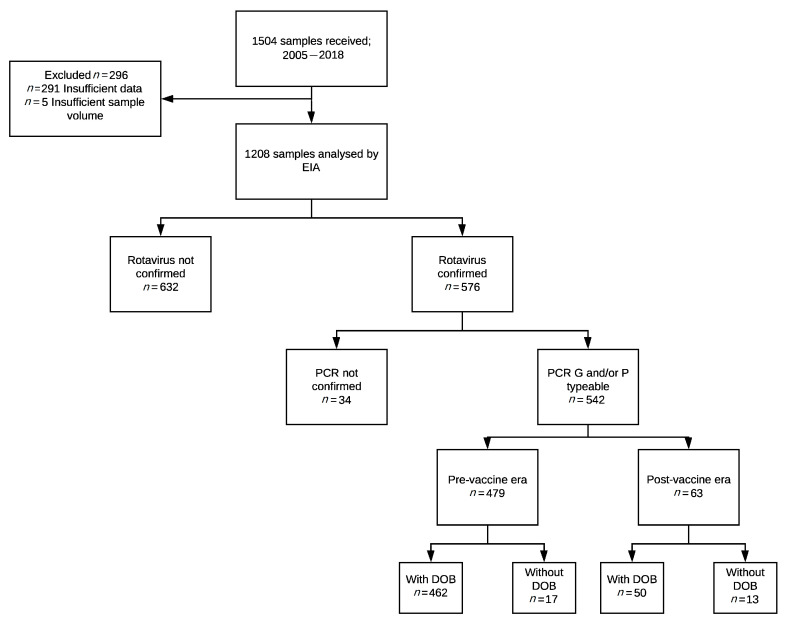
Consort diagram of samples included in this study.

During the study period 2005–2018, a total of 1504 stool samples was collected and sent to the WHO Rotavirus Regional Reference Laboratory (RRL) at the Murdoch Children’s Research Institute (MCRI). Of these, 1208 samples had sufficient data available on the date of collection and stool volume to enable analysis. Of the 1208 samples, a total of 576 were confirmed as rotavirus positive and proceeded to genotype characterisation (Figure 1). Thirty-four samples were not genotyped due to laboratory error, comprising 1 sample from 2010 and 33 samples from 2011, and were subsequently excluded from further analysis. The remaining 542 samples were proceeded with for further analysis (Figure 1).

### 2.2. Genotype Distribution and the Impact of Vaccine Introduction

In the pre-vaccine period (2005–2012), 58% (479/827) of samples received were confirmed as rotavirus positive, compared to only 18% (63/347) of samples in the post-vaccine era (2013–2018) (Table 1). These values may be affected by sampling changes over the study period. Between 2005 and 2009, only positive samples were received; between 2010 and 2016, all positive and negative samples were received; and from 2017 onward, all positive and 10% of all negatives were sent to MCRI. Overall, between 2005–2018, G1P[8] was the most commonly detected genotype (*n* = 157, 29%), with both G2P[4] (*n* = 155, 29%) and G3P[8] (*n* = 144, 27%) detected at similar frequencies, followed by G12P[8] (*n* = 33, 6%) (Table 1). Other genotypes including G2P[8], G3P[6], G8P[8], G9P[8] and G12P[4] as well as mixed or partially typed samples were infrequently detected (*n* = 1–5, 0.2–3%). However, marked differences were observed following vaccine introduction. Prior to vaccine introduction, genotype dominance varied across years, with G3P[8] dominant in 2006 (*n* = 74, 94%) and 2009 (*n* = 26, 59%), G2P[4] dominant in 2008 (*n* = 30, 40%) and 2010 (*n* = 88, 74%), and G1P[8] dominant in 2011 (*n* = 127, 85%) and 2012 (*n* = 4, 67%). However, the number of samples available for genotyping was low in 2005 and 2007 and no clear dominant genotype could be determined.

**Figure 2:**
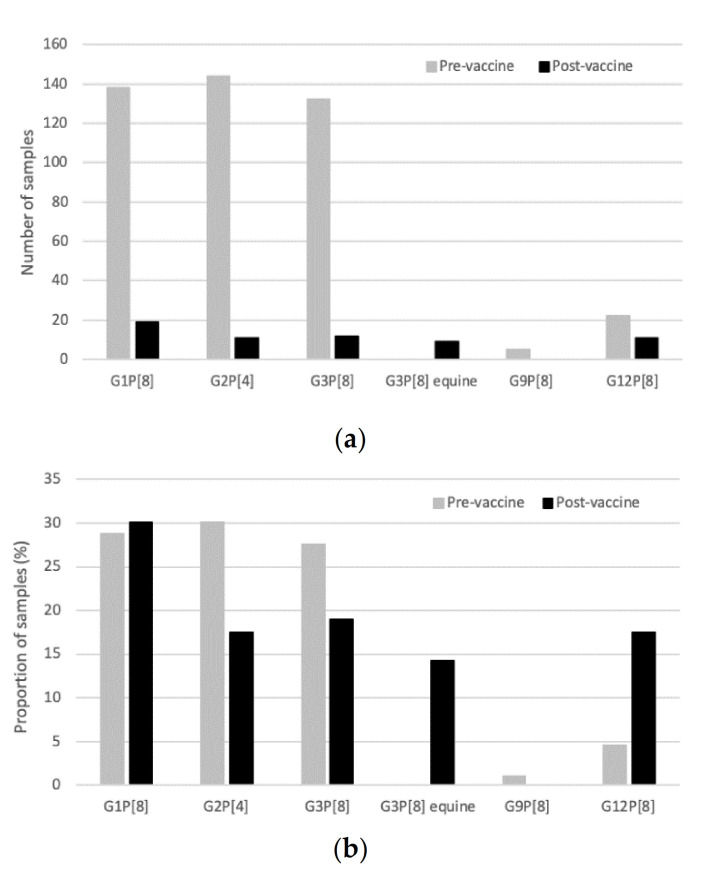
Distribution of main genotypes in samples collected in the pre-vaccine (2005–2012) and post-vaccine (2013–2018) period. (**a**) Number of samples in each of the main genotype groups. (**b**) Proportion of samples identified in each of the main genotype groups, of the total number of samples genotyped.

Following vaccine introduction, there was a marked decrease in the number of rotavirus positive samples available for genotyping, reflecting the reduction in rotavirus disease observed (Table 1). The diversity of genotypes decreased following vaccine introduction (Figure 2) with some genotypes (G2P[8], G3P[6], G8P[8], G9P[8], G12P[4]) no longer detected. There was only one mixed genotype sample identified in the post-vaccine era, compared with 13 mixed genotype samples detected in the pre-vaccine era. The dominant genotype continued to vary annually following vaccine introduction, with G3P[8] dominant in 2013 (*n* = 6, 50%), G1P[8] in 2014 (*n* = 17, 71%), G12P[8] in 2017 (*n* = 11, 100%) and G3P[8] in 2018 (*n* = 6, 100%). The previously dominant G1P[8] disappeared 3 years after vaccine introduction, and G2P[4] strains were not detected after 2014 (Table 1). Emergence of the novel, equine-like G3P[8] reassortant strain, previously not detected in Fiji, was reported in the years following vaccine introduction. This equine-like G3P[8] was dominant for two consecutive years (2015–2016), accounting for 83% (*n* = 5/6) and 100% (*n* = 4/4) of samples genotyped. However, it was not detected in 2017 or 2018. G3P[8] re-emerged in 2018 after not being detected for 4 years.

## 3. Discussion

This is the first study in a low- or middle-income country in the Western Pacific Region to describe rotavirus genotypes following national rotavirus vaccine introduction. Prior to rotavirus vaccine introduction, G1P[8], G2P[4] and G3P[8] were the predominant genotypes causing rotavirus diarrhoea in children less than 5 years of age in Fiji. Genotype diversity decreased following rotavirus vaccine introduction in Fiji; with G2P[8], G3P[6], G8P[8], G9P[8] and G12P[4], which all represented minor genotypes in the pre-vaccine period, subsequently undetected in the vaccine era. Following rotavirus vaccine introduction, G2P[4] has not been detected since 2014 and G1P[8] has not been detected since 2015. This is in contrast to changes in genotype distribution observed in Australia following introduction of the Rotarix (GlaxoSmithKline, Rixensart, Belgium) and RotaTeq (Merck, Kenalworth, NJ, USA) vaccines. In Australia, although there was an overall decrease in the common genotypes (G1, G2, G3, G4 and G9) from 83% to 63% observed following rotavirus vaccine introduction, an increase in G2P[4] (pre-vaccine era 5%; post-vaccine era 21%) was observed in states and territories implementing the Rotarix vaccine and G1P[8] continued to be detected [5].

We found that equine-like G3P[8] was the dominant genotype in 2015 to 2016 but was not detected in the following years (2017 or 2018). An increase in novel zoonotic strains such as equine-like G3P[8] following the introduction of Rotarix has also been observed in other countries (Australia, Japan, Hungary and Brazil) [12,13,14,15,16]. The segmented rotavirus genome allows reassortment to occur both within and between human and animal strains if the human host is infected with two different rotavirus strains, thus giving rise to novel and unusual genotype combinations [17]. In Australia, an increase in G12P[8], equine-like G3P[8], G8, G10 and other zoonotic reassortant strains has also been observed following rotavirus vaccine introduction [5]. In Fiji, human G3P[8] was not detected during 2015 and 2016 when the equine-like G3P[8] was circulating, but this strain re-emerged two years later when equine-like G3P[8] was no longer detected. This is consistent with reports from Asia, Australia, Europe and the U.S. [5,17].

The G12 genotype was first identified in Fiji in 2008, with both G12P[4] and G12P[8] detected. These strains accounted for 32% (*n* = 24/75) of all rotavirus positive samples in 2008 but were not detected again until 2017 when all available samples (*n* = 11) were identified as G12P[8] (Table 1). The emergence of G12 following vaccine introduction has been observed in other countries but does not appear to be dependent on vaccine coverage. In Finland, a 9% increase in G12P[8] was observed five years after vaccine introduction, with a higher frequency of G12P[8] detected in vaccinated children (14%) than observed in unvaccinated children (7%) [18]. In Australia, a small G12P[8] outbreak was reported in 2005 prior to vaccine introduction; however, since vaccine introduction G12P[8] has become common, detected in 18% of samples from children less than 5 years with acute diarrhoea [5]. Similarly, G12, originally detected in Brazil in 2008 following vaccine introduction (2006), has emerged to be the most prevalent genotype (G12P[8]) in 87% of samples in 2014 [19].

A key strength of this study is the ability to observe genotypic changes over time, following the introduction of Rotarix into a national program in a Pacific nation associated with very high vaccine coverage. Monitoring rotavirus genotypes that continue to cause diarrhoea in children provides critical information regarding the ongoing effectiveness of the vaccine program and can assist in outbreak investigation. It also enables early identification of the emergence or importation of new strains that may have a public health impact. This is particularly relevant for Fiji as an island nation with an economy highly dependent on tourism where there is potential for importation of novel strains resulting in disease outbreaks.

This study aligns with data on the impact of rotavirus vaccines on rotavirus disease hospitalisations in children less than 5 years of age in Fiji. Fiji has been notable within the Pacific as a country that has introduced new vaccines based on local data and is committed to monitoring vaccine impact. No other Pacific nation participates in WHO rotavirus surveillance. Data from Fiji may assist in informing vaccine decisions of neighbouring countries in the region. A limitation of this study is that it can only report on samples received for analysis by the WHO Rotavirus Regional Reference Laboratory. Despite attempts, not all children admitted to hospital with diarrhoea have a stool sample collected and sent for analysis. Following introduction of a rotavirus vaccine, the number of children hospitalised with rotavirus disease has dramatically decreased; as a result, the number of stool samples available to provide comparisons of genotypic distribution between the pre-vaccine and post-vaccine era has been impacted. As stool collection is still requested for hospitalised patients with acute diarrhoea in Fiji, it is unlikely that there is a bias impacting on stool collection between the period before and after introduction of the rotavirus vaccine.

The variation in the proportion of rotavirus negative samples reported reflects differences in the rotavirus detection status of stool samples submitted to MCRI for genotypic analysis over the 14-year surveillance period (Table 1). The lower proportion of rotavirus negative samples early in the surveillance period (2005–2009) has limited impact on the outcome of this paper given the focus is the period following the introduction of the rotavirus vaccine.

In 2015, there was a marked increase in the number of negative samples tested. From 2010 to 2013, there was an increase in typhoid detection as the result of six typhoid outbreaks in Fiji, along with outbreaks of both Zika virus and Chikungunya virus, which were both initially detected in 2015, all which may have led to an increased number of negative samples being sent to MCRI for analysis during this time period [20,21,22]. Being negative samples only, this also would have had minimal impact on the rotavirus distribution observed in this study.

The effect of age on rotavirus detection in the stool following introduction of rotavirus vaccines in Fiji has recently been reported [11]. Due to the success of the rotavirus vaccination program, the ability to compare age related differences in genotype distribution in samples from the pre- and post-vaccine eras has been impacted by the limited number of samples available for analysis in the post-vaccine era (pre-vaccine era *n* = 462; post-vaccine era *n* = 50). This decline in number of available rotavirus positive samples was not likely to be due to a lack of sampling due to the ongoing surveillance program operating in Fiji.

In this study we report changes in the pattern of rotavirus genotypes causing diarrhoea in children in Fiji since rotavirus vaccine introduction. We observed a transient increase in the zoonotic strain equine-like G3P[8] and a reduction in the previously dominant G1P[8] and G2P[4]. A decrease in detection of rotavirus strains G2P[8], G3P[6], G8P[8] and G9P[8] was also detected in the years following rotavirus vaccine introduction. Monitoring rotavirus genotypes provides key information regarding the ongoing effectiveness of the vaccine program and can assist in outbreak investigation. It also enables early identification of the emergence or importation of new strains that may have a public health impact.

## 4. Materials and Methods

### 4.1. Population and Study Sites

Fiji has participated in the WHO Global Rotavirus Surveillance Program since 2006, monitoring rotavirus disease burden and rotavirus genotype diversity associated with hospitalisations in children less than 5 years of age. The samples from children hospitalised with acute diarrhoea are sent to the WHO Rotavirus Regional Reference Laboratory at Murdoch Children’s Research Institute (MCRI) according to the case definitions and methods defined for the WHO Global Rotavirus Surveillance Program [11]. Two major hospitals admitting children with gastroenteritis in Fiji participated in this study. The Colonial War Memorial Hospital (CWMH) is Fiji’s largest general hospital and is the main referral centre for the greater Suva area with approximately 34,920 children under 5 years of age. The Savusavu District Hospital is a secondary health inpatient and outpatient facility serving mainly a semiurban and rural population with an estimated 6563 children under 5 years of age. Samples were received from inpatients at Savusavu district hospital only in 2013 and 2014. Details on rotavirus surveillance in Fiji has previously been described [9,11].

### 4.2. Participants

A prospective rotavirus surveillance program was established in 2005 to effectively capture rotavirus detected in faecal samples from children less than 5 years with acute nonbloody diarrhoea in Fiji. Acute diarrhoea was defined as 3 or more loose, nonbloody stools within a 24-h period for <14 days. Eligible participants were identified by checking admission data and children’s wards daily, parental/guardian consent was obtained, and stool was collected within 48 h of admission. Once rotavirus positivity was determined, demographics and clinical information were obtained via medical records.

Ethics approval for these studies was obtained from the Fiji National Research Ethics Review Committee (number 2013-40) and from the University of Melbourne Human Research Ethics Committee for the initial study surveillance in Colonial War Memorial Hospital from 2005–2012 (Ethics ID:050546X) and Savusavu from 2010–2012 (Ethics ID:0931282); during this period written informed consent was obtained from participants’ parents. From June 2012 onward, the Ministry of Health and Medical Services considered this public health surveillance and no longer required written consent.

### 4.3. Genotyping

Faecal specimens were collected and stored at 4–8 °C prior to being transported to the Fiji Centre for Communicable Disease Control in Suva for rotavirus antigen testing via the ProSpecT Rotavirus test, a commercial rotavirus enzyme immunoassay (EIA) (Thermofisher Scientific, Waltham, MA, USA) as per manufacturer’s instructions. Stool samples were then stored at −70 °C. De-identified rotavirus specimens were transported on dry ice to the WHO Rotavirus Regional Reference Laboratory at the Murdoch Children’s Research Institute, Parkville, Australia. Sample selection for shipment to MCRI varied during the surveillance program. Between 2005 and 2009 only stool samples that tested positive to rotavirus in the Fiji laboratory were sent to MCRI, with the negative samples reflected in Table 1 having been identified by EIA at MCRI. Between 2010 and 2016, stool samples were sent to MCRI for EIA and RT-PCR genotyping irrespective of whether they were rotavirus positive or negative via EIA conducted in Fiji. From 2017 onward, all rotavirus positive samples and 10% of rotavirus negative samples by EIA in Fiji were sent to MCRI for further analysis. All samples were retested at MCRI to confirm rotavirus positivity prior to proceeding with genotypic analysis. Rotavirus positivity (or negativity) was confirmed using the ProSpecT Rotavirus test, (EIA) (Thermofisher Scientific, Waltham, MA, USA) as per the manufacturer’s instructions. Stool samples that tested positive or equivocal for rotavirus antigen were further characterised to determine the G and P genotype. Samples from 2005 and 2006 were routinely serotyped using an in-house monoclonal antibody based serotyping EIA. This EIA consisted of a panel of monoclonal antibodies specific to the VP7 outer capsid protein of group A rotavirus serotypes G1, G2, G3, G4 and G9 [23]. Prior to 2007, P-typing and RT-PCR were not routinely performed. From 2007 onward, rotavirus G and P genotypes were determined by heminested multiplex RT-PCR assay. All samples collected prior to 2007 have retrospectively been characterised by heminested RT-PCR G and P genotyping. In brief, viral RNA was extracted from 20% (*w*/*v*) faecal extracts in a virus dilution buffer (0.01 M Tris-HCL [pH7.5], 10.5 mM CaCl, 145 mM NaCl) using the QIAamp Viral RNA mini extraction kit (QIAGEN, Hilden, Germany) according to the manufacturer’s instructions.

The One-step RT-PCR kit (QIAGEN) was used to perform first round PCR, using VP7 primers VP7F and VP7R and VP4 primers VP4F and VP4R [24,25]. Second round genotyping PCR was performed using AmpliTaq DNA Polymerase with Buffer II (Applied Biosystems, Foster City, CA, USA), with specific G and P oligonucleotide primers for G typing (G1, G2, G3, G4, G8 and G9) or P typing (P[4], P[6], P[8], P[9], P[10] and P[11]) as described previously [26]. Amplified products were run on a 1.5% or 2% agarose gel for G and P types respectively, and genotypes were determined based on amplicon band size. PCR non-typeable samples were determined by Sanger sequencing. Strains including equine-like G3, G12 and unusual or uncommon strains were unable to be genotyped using standard primers. VP7 or VP4 amplicons from first round PCR products were purified for sequencing using the Wizard SV Gel and PCR Clean up System (Promega, Madison, WI, USA) as per manufacturer’s protocol. Purified DNA with oligonucleotide primers (VP7F/R or VP4F/R) were sent to the Australian Genome Research Facility (AGRF) Melbourne and sequenced using an ABI PRISM BigDye Terminator Cycle Sequencing Reaction Kit (Applied Biosystems, Foster City, CA, USA) in an Applied Biosystems 3730xl DNA analyser. Sequencher version 4.10.1 (Gene Codes Corporation, Ann Arbor, MI, USA) was used to edit the sequences. BLAST (http://blast.ncbi.nlm.nih.gov/Blast.cgi (accessed on 10 October 2020)) and RotaC version 2.0 (http://rotac.regatools.be (accessed on 10 October 2020)) [27] were used determine the genotype of each sample.

### 4.4. Data Analysis

Samples were excluded if there was no date of stool collection available, if there was insufficient sample to process, if the sample was not confirmed as rotavirus positive by EIA at MCRI, or if samples were rotavirus positive by EIA at MCRI but genotype could not be determined. To describe the impact of rotavirus vaccine introduction on genotype distribution, samples were grouped into pre-vaccine (2005–2012) and post-vaccine (2013–2018) eras according to the date of collection. Analysis is by descriptive observations and comparisons between the pre-vaccine and post-vaccine introduction eras.

## Figures and Tables

**Table 1:** Genotype distribution in samples received by the WHO Regional Reference Laboratory.

	G1P[8]		G2P[4]		G2P[8]		G3P[6]		G3P[8]		G3P[8] EQUINE		G8P[8]		G9P[8]		G12P[4]		G12P[8]		Mixed		Partially Typed		Total Genotyped		Negative		Total Samples
Year	*n*	%	*n*	%	*n*	%	*n*	%	*n*	%	*n*	%	*n*	%	*n*	%	*n*	%	*n*	%	*n*	%	*n*	%	*n*	%	*n*	%	*n*
2005	1	20							1	20					1	20							2	40	5	71	2	29	7
2006							1	1	74	94													4	5	79	99	1	1	80
2007									1	50													1	50	2	100	0	0.0	2
2008	5	7	30	40	4	5			1	1					3	4	2	3	22	29	8	11			75	100	0	0.0	75
2009			9	21					26	59			1	2	1	2					2	5	5	11	44	96	2	4	46
2010	1	1	88	74					23	19											3	3	4	3	119	62	74	38	193
2011	127	85	15	10					6	4													1	1	149	37	255	63	404
2012	4	67	2	33																					6	30	14	70	20
Subtotal	138		144		4		1		132		0		1		5		2		22		13		17		479		348		827
Rotarix Vaccine Introduced																													
2013	1	8	5	42					6	50															12	67	6	33	18
2014	17	71	6	25																	1	4			24	51	23	49	47
2015	1	17									5	83													6	4	158	96	164
2016											4	100													4	15	22	85	26
2017																			11	100					11	20	43	80	54
2018									6	100															6	16	32	84	38
Subtotal	19		11		0		0		12		9		0		0		0		11		1		0		63		284		347

## Data Availability

Data is contained within the article.
